# Discordance between MRI and echocardiography in the assessment of mitral regurgitation severity: prediction of surgical response suggests mri may be more accurate

**DOI:** 10.1186/1532-429X-17-S1-P262

**Published:** 2015-02-03

**Authors:** Seth Uretsky, Edgar Argulian, Sai Priyanka Gudiwada, Randy Cohen, James Jang, Farooq A Chaudhry, Leo Marcoff, Konstantinos Koulogiannis, Linda D Gillam, Steven Wolff

**Affiliations:** Cardiovascular Medicine, Morristown Medical Center, Morristown, NJ USA; St. Luke’s and Roosevelt Hospitals, New York, NY USA; Department of Medicine, San Jose Medical Center, San Jose, CA USA; Carnegie Hill Radiology, New York, NY USA; Cardiovascular Medicine, Mount Sinai School of Medicine, Kragujevac, NY USA

## Background

The assessment of mitral regurgitation (MR) severity is often based on echocardiographic criteria and clinical assessment. MRI can also quantify MR severity, indirectly as the difference between left ventricular (LV) stroke volume and forward flow. In clinical practice these imaging modalities can be discordant. The purpose of this prospective, multicenter trial is to compare the two techniques in assessing the severity of MR.

## Methods

To date, this IRB approved trial includes 103 patients (61 ± 14yrs, male 57%) with MR (19 functional, 49 degenerative, 35 other). All patients had 2D transthoracic echocardiography (Echo) and 39 (38%) patients had a transesophageal Echo. The studies were generally complete and allowed for both a qualitative and quantitative assessment of mitral regurgitant severity by experienced echocardiographers blinded to the MRI. Using the integrated approach advocated by the American Society of Echocardiography, mitral regurgitant severity was qualitatively characterized as mild, moderate, or severe. In addition, echocardiographic regurgitant volume was quantified by PISA. The same patients underwent MRI to determine MR regurgitant severity, which was calculated as the difference between LV stroke volume and forward flow. LV stroke volume was determined from short axis cine images. Phase-contrast images of the great vessels were used to determine forward flow. MR severity was categorized as mild <30 ml, moderate 30-59 ml, or severe ≥60 ml. A subset of 33 patients (10 mitral valve replacement, 23 repair) had isolated mitral valve surgery, of which 25 returned for a follow-up MRI after 4-6 months. For Echo and MRI, the presurgical MR regurgitant volume was correlated with the postoperative decrease in LV end-diastolic volume (EDV).

## Results

Agreement was poor (37/103=36%, Table) between MRI and the integrated echocardiographic approach to assessing MR severity. Discordance between MRI and Echo was sometimes striking. For example, of the 58 patients that were thought to have severe MR on Echo, 34% were determined to have mild MR by MRI. Similarly poor comparative results were obtained when using purely quantitative Echo parameters such as PISA to quantify regurgitant volume (r^2^=0.3, p<0.001). In the 25 patients that were referred for isolated mitral valve surgery and who returned for follow-up MRI, there was a good correlation between MRI regurgitant volume and decrease in LV EDV (r^2^=0.74, p<0.001, Figure). There was no significant correlation between Echo regurgitant volume and decrease in LV EDV (r^2^=0.1, p-0.1, Figure).

**Table 1:** Comparison of Mitral Regurgitation Severity by MRI and Echocardiography

			MRI	
		Mild	Moderate	Severe
Echo	Mild	14	0	0
	Moderate	19	10	2
	Severe	20	25	13

**Figure 1:**
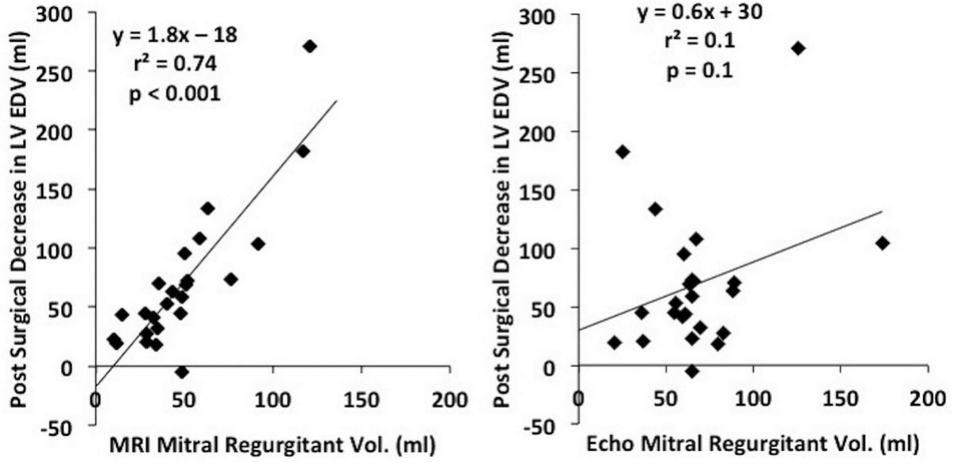
Correlation between mitral regurgitant volume qauntified by MRI and Echo and the post-surgical decrease in LV EDV.

## Conclusions

MRI and echocardiography have only a modest correlation in the assessment of MR severity. The strong correlation between MR severity and post surgical remodeling suggests that MRI is the more accurate test.

